# Supplementary material for: Benzodiazepines and Mood Stabilizers in Schizophrenia Patients Treated with Oral versus Long-Acting Injectable Antipsychotics—An Observational Study
Source: Brain Sci. 2023 Jan 20;13(2):173. doi: 10.3390/brainsci13020173 (PMC9953951; doi:10.3390/brainsci13020173)
Supplement: Supplementary file 1 [file brainsci-13-00173-s001.zip › References_BZD_MS_Final.docx]

References

1. Kahn, R.S.; Sommer, I.E.; Murray, R.M.; Meyer-Lindenberg, A.; Weinberger, D.R.; Cannon, T.D.; O’Donovan, M.; Correll, C.U.; Kane, J.M.; van Os, J.; et al. Schizophrenia. *Nat. Rev. Dis. Primers* **2015**, *1*, 15067. https://doi.org/10.1038/nrdp.2015.67.
2. Johnstone, E.C.; Geddes, J. How high is the relapse rate in schizophrenia? *Acta Psychiatr. Scand. Suppl.* **1994**, *382*, 6–10. https://doi.org/10.1111/j.1600-0447.1994.tb05858.x.
3. Lader, M. What is relapse in schizophrenia? *Int. Clin. Psychopharmacol.* **1995**, *9* (Suppl. 5), 5–9. https://doi.org/10.1097/00004850-199501005-00002.
4. Temmingh, H.; Stein, D.J. Anxiety in Patients with Schizophrenia: Epidemiology and Management. *CNS Drugs* **2015**, *29*, 819–832. https://doi.org/10.1007/s40263-015-0282-7.
5. Enticott, P.G.; Ogloff, J.R.; Bradshaw, J.L. Response inhibition and impulsivity in schizophrenia. *Psychiatry Res.* **2008**, *157*, 251–254. https://doi.org/10.1016/j.psychres.2007.04.007.
6. Xi, S.J.; Shen, M.X.; Wang, Y.; Zhou, W.; Xiao, S.Y.; Tebes, J.K.; Yu, Y. Depressive symptoms, anxiety symptoms, and their co-occurrence among people living with schizophrenia in China: Prevalence and correlates. *J. Clin. Psychol.* **2021**, *77*, 2137–2146. https://doi.org/10.1002/jclp.23141.
7. Correll, C.U.; Citrome, L.; Haddad, P.M.; Lauriello, J.; Olfson, M.; Calloway, S.M.; Kane, J.M. The Use of Long-Acting Injectable Antipsychotics in Schizophrenia: Evaluating the Evidence. *J. Clin. Psychiatry* **2016**, *77* (Suppl. 3),3–24. https://doi.org/10.4088/JCP.15032su1.
8. Taipale, H.; Mittendorfer-Rutz, E.; Alexanderson, K.; Majak, M.; Mehtälä, J.; Hoti, F.; Jedenius, E.; Enkusson, D.; Leval, A.; Sermon, J.; et al. Antipsychotics and mortality in a nationwide cohort of 29,823 patients with schizophrenia. *Schizophr. Res.* **2018**, *197*, 274–280. https://doi.org/10.1016/j.schres.2017.12.010.
9. Kishimoto, T.; Hagi, K.; Kurokawa, S.; Kane, J.M.; Correll, C.U. Long-acting injectable versus oral antipsychotics for the maintenance treatment of schizophrenia: A systematic review and comparative meta-analysis of randomised, cohort, and pre-post studies. *Lancet Psychiatry* **2021**, *8*, 387–404. https://doi.org/10.1016/S2215-0366(21)00039-0.
10. Kane, J.M.; McEvoy, J.P.; Correll, C.U.; Llorca, P.M. Controversies Surrounding the Use of Long-Acting Injectable Antipsychotic Medications for the Treatment of Patients with Schizophrenia. *CNS Drugs* **2021**, *35*, 1189–1205. https://doi.org/10.1007/s40263-021-00861-6.
11. Ifteni, P.; Petric, P.S.; Teodorescu, A. Rating Opportunity for Long-Acting Injectable Antipsychotic Initiation Index (ROLIN). *Front. Psychiatry* **2021**, *12*, 767756. https://doi.org/10.3389/fpsyt.2021.767756.
12. Lehman, A.F.; Lieberman, J.A.; Dixon, L.B.; McGlashan, T.H.; Miller, A.L.; Perkins, D.O.; Kreyenbuhl, J.; American Psychiatric Association; Steering Committee on Practice Guidelines. Practice guideline for the treatment of patients with schizophrenia, second edition. *Am. J. Psychiatry* **2004**, *161* (Suppl. 2), i-iv+1-56.
13. Lähteenvuo, M.; Tiihonen, J. Antipsychotic Polypharmacy for the Management of Schizophrenia: Evidence and Recommendations. *Drugs* **2021**, *81*, 1273–1284. https://doi.org/10.1007/s40265-021-01556-4.
14. Ballon, J.; Stroup, T.S. Polypharmacy for schizophrenia. *Curr. Opin. Psychiatry* **2013**, *26*, 208–213. https://doi.org/10.1097/YCO.0b013e32835d9efb.
15. Baandrup, L. Polypharmacy in schizophrenia. *Basic Clin. Pharmacol. Toxicol.* **2020**, *126*, 183–192. https://doi.org/10.1111/bcpt.13384.
16. Włodarczyk, A.; Szarmach, J.; Cubała, W.J.; Wiglusz, M.S. Benzodiazepines in combination with antipsychotic drugs for schizophrenia: GABA-ergic targeted therapy. *Psychiatr. Danub.* **2017**, *29* (Suppl. 3), 345–348.
17. Szarmach, J.; Włodarczyk, A.; Cubała, W.J.; Wiglusz, M.S. Benzodiazepines as adjunctive therapy in treatment refractory symptoms of schizophrenia. *Psychiatr. Danub.* **2017**, *29* (Suppl. 3), 349–352.
18. Stimmel, G.L. Benzodiazepines in schizophrenia. *Pharmacotherapy* **1996**, *16 Pt 2*, 148S–151S; discussion 166S–168S.
19. Van Kammen, D.P. gamma-Aminobutyric acid (Gaba) and the dopamine hypothesis of schizophrenia. *Am. J. Psychiatry* **1977**, *134*, 138–143. https://doi.org/10.1176/ajp.134.2.138.
20. Sim, F.; Sweetman, I.; Kapur, S.; Patel, M.X. Re-examining the role of benzodiazepines in the treatment of schizophrenia: A systematic review. *J. Psychopharmacol.* **2015**, *29*, 212–223. https://doi.org/10.1177/0269881114541013.
21. Strømme, M.F.; Mellesdal, L.S.; Bartz-Johannesen, C.A.; Kroken, R.A.; Krogenes, M.L.; Mehlum, L.; Johnsen, E. Use of Benzodiazepines and Antipsychotic Drugs Are Inversely Associated with Acute Readmission Risk in Schizophrenia. *J. Clin. Psychopharmacol.* **2022**, *42*, 37–42. https://doi.org/10.1097/JCP.0000000000001497.
22. Fontanella, C.A.; Campo, J.V.; Phillips, G.S.; Hiance-Steelesmith, D.L.; Sweeney, H.A.; Tam, K.; Lehrer, D.; Klein, R.; Hurst, M. Benzodiazepine use and risk of mortality among patients with schizophrenia: A retrospective longitudinal study. *J. Clin. Psychiatry* **2016**, *77*, 661–667. https://doi.org/10.4088/JCP.15m10271.
23. Citrome, L. Treatment-resistant schizophrenia: What role for mood stabilizers? *Curr. Psychiatry* **2004**, *3*, 23–40.
24. Chiu, C.-T.; Wang, Z.; Hunsberger, J.G.; Chuang, D.-M. Therapeutic Potential of Mood Stabilizers Lithium and Valproic Acid: Beyond Bipolar Disorder. *Pharmacol. Rev.* **2013**, *65*, 105–142. https://doi.org/10.1124/pr.111.005512.
25. Leucht, S.; Helfer, B.; Dold, M.; Kissling, W.; McGrath, J. Lithium for schizophrenia. *Cochrane Database Syst. Rev.* **2015**, *10*, CD003834. https://doi.org/10.1002/14651858.CD003834.pub3.
26. Citrome, L.; Levine, J.; Allingham, B. Changes in use of valproate and other mood stabilizers for patients with schizophrenia from 1994 to 1998. *Psychiatr. Serv.* **2000**, *51*, 634–638. https://doi.org/10.1176/appi.ps.51.5.634.].
27. Puranen, A.; Koponen, M.; Tanskanen, A.; Tiihonen, J.; Taipale, H. Use of antidepressants and mood stabilizers in persons with first-episode schizophrenia. *Eur. J. Clin. Pharmacol.* **2020**, *76*, 711–718. https://doi.org/10.1007/s00228-020-02830-2.
28. Lim, W.K.; Chew, Q.H.; He, Y.L.; Si, T.M.; Chiu, F.H.; Xiang, Y.T.; Kato, T.A.; Kanba, S.; Shinfuku, N.; Lee, M.S.; et al. Coprescription of mood stabilizers in schizophrenia, dosing, and clinical correlates: An international study. *Hum. Psychopharmacol.* **2020**, *35*, 1–7. https://doi.org/10.1002/hup.2752.
29. Berle, J.Ø.; Spigset, O. Har stemningsstabiliserende legemidler noen plass i behandlingen av schizofreni? [Are mood stabilizers beneficial in the treatment of schizophrenia?]. *Tidsskr Nor Laegeforen.* **2005**, *125*, 1809–1812.
30. Casey, D.E.; Daniel, D.G.; Wassef, A.A.; Tracy, K.A.; Wozniak, P.; Sommerville, K.W. Effect of divalproex combined with olanzapine or risperidone in patients with an acute exacerbation of schizophrenia. *Neuropsychopharmacology.* **2003**, *28*, 182–192. https://doi.org/10.1038/sj.npp.1300023.
31. Puranen, A.; Koponen, M.; Lähteenvuo, M.; Tanskanen, A.; Tiihonen, J.; Taipale, H. Real-world effectiveness of mood stabilizer use in schizophrenia. *Acta Psychiatr. Scand.* 2022, *ahead of print*. https://doi.org/10.1111/acps.13498.
32. Tseng, P.T.; Chen, Y.W.; Chung, W.; Tu, K.Y.; Wang, H.Y.; Wu, C.K.; Lin, P.Y. Significant Effect of Valproate Augmentation Therapy in Patients with Schizophrenia: A Meta-analysis Study. *Medicine* **2016**, *95*, e2475. https://doi.org/10.1097/MD.0000000000002475.
33. Atkins, M.; Burgess, A.; Bottomley, C.; Riccio, M. Chlorpromazine equivalents: A consensus of opinion for both clinical and research applications. *Psychiatr. Bull.* **1997**, *21*, 224–226. https://doi.org/10.1192/pb.21.4.224.
34. Inada, T., Inagaki, A. Psychotropic dose equivalence in Japan. Psychiatry Clin Neurosci. 2015 Aug;69(8):440-7. doi: 10.1111/pcn.12275. Epub 2015 Feb 14. PMID: 25601291.
35. Gardner, D.M.; Murphy, A.L.; O’Donnell, H.; Centorrino, F.; Baldessarini, R.J. International consensus study of antipsychotic dosing. *Am. J. Psychiatry* **2010**, *167*, 686–693.
36. Leucht, S.; Samara, M.; Heres, S.; Patel, M.X.; Furukawa, T.; Cipriani, A.; Geddes, J.; Davis, J.M. Dose Equivalents for Second-Generation Antipsychotic Drugs: The Classical Mean Dose Method. *Schizophr. Bull.* **2015**, *41*, 1397–1402. https://doi.org/10.1093/schbul/sbv037.
37. Sajatovic, M.; Ross, R.; Legacy, S.N.; Correll, C.U.; Kane, J.M.; DiBiasi, F.; Fitzgerald, H.; Byerly, M. Identifying patients and clinical scenarios for use of long-acting injectable antipsychotics—Expert consensus survey part 1. *Neuropsychiatr. Dis. Treat.* **2018**, *14*, 1463–1474. https://doi.org/10.2147/NDT.S167394.
38. Offord, S.; Wong, B.; Mirski, D.; Baker, R.A.; Lin, J. Healthcare resource usage of schizophrenia patients initiating long-acting injectable antipsychotics vs oral. *J. Med. Econ.* **2013**, *16*, 231–239. https://doi.org/10.3111/13696998.2012.751025.
39. Bareis, N.; Olfson, M.; Wall, M.; Stroup, T.S. Variation in Psychotropic Medication Prescription for Adults with Schizophrenia in the United States. *Psychiatr. Serv.* 2022 May;73(5):492-500. doi: 10.1176/appi.ps.202000932. Epub 2021 Sep 30. PMID: 34587788; PMCID: PMC8964836.
40. Reymann, S.; Schoretsanitis, G.; Egger, S.T.; Mohonko, A.; Kirschner, M.; Vetter, S.; Homan, P.; Seifritz, E.; Burrer, A. Use of Long-Acting Injectable Antipsychotics in Inpatients with Schizophrenia Spectrum Disorder in an Academic Psychiatric Hospital in Switzerland. *J. Pers. Med.* **2022**, *12*, 441. https://doi.org/10.3390/jpm12030441.
41. Stip, E.; Lachaine, J. Real-world effectiveness of long-acting antipsychotic treatments in a nationwide cohort of 3957 patients with schizophrenia, schizoaffective disorder and other diagnoses in Quebec. *Ther. Adv. Psychopharmacol.* **2018**, *8*, 287–301; Erratum in *Ther. Adv. Psychopharmacol.* **2018**, *8*, 327. https://doi.org/10.1177/2045125318782694.
42. Pilon, D.; Joshi, K.; Tandon, N.; Lafeuille, M.H.; Kamstra, R.L.; Emond, B.; Lefebvre, P. Treatment patterns in Medicaid patients with schizophrenia initiated on a first- or second-generation long-acting injectable versus oral antipsychotic. *Patient Prefer. Adherence* **2017**, *11*, 619–629. https://doi.org/10.2147/PPA.S127623.
43. Llorca, P.M.; Bobes, J.; Fleischhacker, W.W.; Heres, S.; Moore, N.; Bent-Ennakhil, N.; Sapin, C.; Loze, J.Y.; Nylander, A.G.; Patel, M.X. Baseline results from the European non-interventional Antipsychotic Long acTing injection in schizOphrenia (ALTO) study. *Eur. Psychiatry* **2018**, *52*, 85–94. https://doi.org/10.1016/j.eurpsy.2018.04.004.
44. Martin, A.; Bessonova, L.; Hughes, R.; Doane, M.J.; O’Sullivan, A.K.; Snook, K.; Cichewicz, A.; Weiden, P.J.; Harvey, P.D. Systematic Review of Real-World Treatment Patterns of Oral Antipsychotics and Associated Economic Burden in Patients with Schizophrenia in the United States. *Adv. Ther.* **2022**, *39*, 3933–3956. https://doi.org/10.1007/s12325-022-02232-z.
45. Lally, J.; Gaughran, F.; Timms, P.; Curran, S.R. Treatment-resistant schizophrenia: Current insights on the pharmacogenomics of antipsychotics. *Pharm. Pers. Med.* **2016**, *9*, 117. https://doi.org/10.2147/PGPM.S115741.
46. Miron, A.A.; Ifteni, P.I.; Teodorescu, A.; Petric, P.S. Long-Acting Injectable Antipsychotics (LAIs) Prescribing Trends during the COVID-19 Pandemic in Romania. *Healthcare* **2022**, *10*, 1265. https://doi.org/10.3390/healthcare10071265.
47. Miron, A.A.; Teodorescu, A.; Ifteni, P.; Irimie, C.A.; Dima, L.; Petric, P.S. Switch from Olanzapine Long-Acting Injectable to its Oral Equivalent during COVID-19 Pandemic: A Real World Observational Study. *Psychiatr. Q.* **2022**, *93*, 627–635. https://doi.org/10.1007/s11126-021-09924-9.
48. Gruppuso, P.A.; Ahmed, R.; Adashi, E.Y. Valproate Teratogenicity: A Moving Target. *Obstet. Gynecol.* **2022**, *140*, 408–411. https://doi.org/10.1097/AOG.0000000000004893.
49. Paton, C.; Citrome, L.; Fernandez-Egea, E.; Rendora, O.; Barnes, T.R.E. Who is prescribed valproate and how carefully is this treatment reviewed in UK mental health services? Data from a clinical audit. *Ther. Adv. Psychopharmacol.* **2022**, *12*,, 1-15. https://doi.org/10.1177/20451253221110016.
50. Wang, Y.; Xia, J.; Helfer, B.; Li, C.; Leucht, S. Valproate for schizophrenia. *Cochrane Database Syst Rev.* **2016**, *2016*, CD004028. https://doi.org/10.1002/14651858.CD004028.pub4.
51. Leucht, S.; Helfer, B.; Dold, M.; Kissling, W.; McGrath, J. Carbamazepine for schizophrenia. *Cochrane Database Syst. Rev*. **2014**, *2014*, CD001258. https://doi.org/10.1002/14651858.CD001258.pub3.
52. Premkumar, T.S.; Pick, J. Lamotrigine for schizophrenia. *Cochrane Database Syst. Rev.* **2006**, *2006*, CD005962. https://doi.org/10.1002/14651858.CD005962.pub2.
53. Paton, C.; Banham, S.; Whitmore, J. Benzodiazepines in schizophrenia: Is there a trend towards long-term prescribing? *Psychiatr. Bull.* **2000**, *24*, 113–115. https://doi.org/10.1192/pb.24.3.113.
54. Dold, M.; Li, C.; Gillies, D.; Leucht, S. Benzodiazepine augmentation of antipsychotic drugs in schizophrenia: A meta-analysis and Cochrane review of randomized controlled trials. *Eur. Neuropsychopharmacol.* **2013**, *23*, 1023–1033. https://doi.org/10.1016/j.euroneuro.2013.03.001.
55. Dold, M.; Li, C.; Tardy, M.; Khorsand, V.; Gillies, D.; Leucht, S. Benzodiazepines for schizophrenia. *Cochrane Database Syst. Rev.* 2012, 11, CD006391. https://doi.org/10.1002/14651858.CD006391.pub2.
56. Stewart, S.A. The effects of benzodiazepines on cognition. *J. Clin. Psychiatry* **2005**, *66* (Suppl. 2) 9–13.
57. Nielsen, S. Benzodiazepines. *Curr. Top. Behav. Neurosci.* **2017**, *34*, 141–159. https://doi.org/10.1007/7854_2015_425.
58. Marcum, Z.A.; Gellad, W.F. Medication adherence to multidrug regimens. *Clin. Geriatr. Med.* **2012**, *28*, 287–300. https://doi.org/10.1016/j.cger.2012.01.008.
59. Leucht, S.; Crippa, A.; Siafis, S.; Patel, M.X.; Orsini, N.; Davis, J.M. Dose-Response Meta-Analysis of Antipsychotic Drugs for Acute Schizophrenia. *Am. J. Psychiatry* **2020**, *177*, 342–353; Erratum in *Am. J. Psychiatry* **2020**, *177*, 272. https://doi.org/10.1176/appi.ajp.2019.19010034.
60. Sparshatt, A.; Jones, S.; Taylor, D. Quetiapine: Dose-response relationship in schizophrenia. *CNS Drugs* **2008**, *22*, 49–68; discussion 69–72. https://doi.org/10.2165/00023210-200822010-00004.
61. Armitage, R.; Cole, D.; Suppes, T.; Ozcan, M.E. Effects of clozapine on sleep in bipolar and schizoaffective disorders. *Prog. Neuropsychopharmacol. Biol. Psychiatry* **2004**, *28*, 1065–1070. https://doi.org/10.1016/j.pnpbp.2004.05.048.
62. Ali, T.; Sisay, M.; Tariku, M.; Mekuria, A.N.; Desalew, A. Antipsychotic-induced extrapyramidal side effects: A systematic review and meta-analysis of observational studies. *PLoS ONE* **2021**, *16*, e0257129. https://doi.org/10.1371/journal.pone.0257129.
63. Novick, D.; Haro, J.M.; Bertsch, J.; Haddad, P.M. Incidence of extrapyramidal symptoms and tardive dyskinesia in schizophrenia: Thirty-six-month results from the European schizophrenia outpatient health outcomes study. *J. Clin. Psychopharmacol.* **2010**, *30*, 531–540. https://doi.org/10.1097/JCP.0b013e3181f14098.
64. Park, S.C.; Choi, M.Y.; Choi, J.; Park, E.; Tchoe, H.J.; Suh, J.K.; Kim, Y.H.; Won, S.H.; Chung, Y.C.; Bae, K.Y.; et al. Comparative Efficacy and Safety of Long-acting Injectable and Oral Second-generation Antipsychotics for the Treatment of Schizophrenia: A Systematic Review and Meta-analysis. *Clin. Psychopharmacol. Neurosci.* **2018**, *16*, 361–375. https://doi.org/10.9758/cpn.2018.16.4.361.
65. Miller, D.D. Atypical antipsychotics: Sleep, sedation, and efficacy. *Prim. Care Companion J. Clin. Psychiatry* **2004**, *6* (Suppl. 2), 3–7.
66. Wong, J.; Delva, N. Clozapine-induced seizures: Recognition and treatment. *Can. J. Psychiatry* **2007**, *52*, 457–463. https://doi.org/10.1177/070674370705200708.
67. Lin, S.K. Antipsychotic Polypharmacy: A Dirty Little Secret or a Fashion? *Int. J. Neuropsychopharmacol.* **2020**, *23*, 125–131. https://doi.org/10.1093/ijnp/pyz068.
68. Tiihonen, J.; Taipale, H.; Mehtälä, J.; Vattulainen, P.; Correll, C.U.; Tanskanen, A. Association of Antipsychotic Polypharmacy vs Monotherapy With Psychiatric Rehospitalization Among Adults With Schizophrenia. *JAMA Psychiatry* **2019**, *76*, 499–507. https://doi.org/10.1001/jamapsychiatry.2018.4320.
69. Zhang, L.; He, S.; He, L.; Yu, W.; He, S.; Li, Y.; Yu, Y.; Zheng, Q.; Huang, J.; Shen, Y.; et al. Long-Term Antipsychotic Effectiveness and Comparison of the Efficacy of Monotherapy and Polypharmacy in Schizophrenia: A 3-Years Follow-Up “Real World” Study in China. *Front. Pharmacol.* **2022**, *13*, 860713. https://doi.org/10.3389/fphar.2022.860713.
