# Supplementary material for: Benzodiazepines and Mood Stabilizers in Schizophrenia Patients Treated with Oral versus Long-Acting Injectable Antipsychotics—An Observational Study
Source: Brain Sci. 2023 Jan 20;13(2):173. doi: 10.3390/brainsci13020173 (PMC9953951; doi:10.3390/brainsci13020173)
Supplement: Supplementary file 1 [file brainsci-13-00173-s001.zip › Table_S1_Demographics and treatment characteristics of the study population.docx]

| Parameters | | LAIs | OAPs | *p*-Value |
| --- | --- | --- | --- | --- |
| **Number of patients (N, %)** | | 77 (24.44%) | 238 (75.56%) | *p* < 0.0001 |
| **Male gender (N, %)** | | 34 (44.15%) | 96 (40.34%) | *p* =0.55 |
| **Mean age (±SD)** | | 52.92 (±12.24 SD) | 51.32 (±11.10 SD) | *p* =0.28 |
| **Patients receiving BZD (N, %)** | Total | 29 (37.66%) | 90 (37.81%) | *p* = 0.98 |
|  | Male | 11 (37.93%) | 34 (37.77%) | *p* = 0.98 |
|  | Female | 18 (62.07%) | 56 (62.23%) | *p* = 0.93 |
| **Patients receiving MS (N, %)** | Total | 16 (20.77%) | 68 (28.57%) | *p* = 0.17 |
|  | Male | 8 (50%) | 32 (47.05%) | *p* = 0.83 |
|  | Female | 8 (50%) | 36 (52.95%) | *p* = 0.83 |
| **Patients receiving both MS and BZD (N, %)** | Total | 10 (12.98%) | 29 (12.18%) | *p* = 0.85 |
|  | Male | 5 (50%) | 15 (51.72%) | *p* = 0.92 |
|  | Female | 5 (50%) | 14 (48.28%) | *p* = 0.92 |
| **Antipsychotic type** | OLZ (N, %) | 5 (6.49%) | 70 (29.41%) | *p* ˂ 0.0001 |
|  | RIS (N, %) | 16 (20.77%) | 31 (13.02%) | *p* = 0.09 |
|  | PAL (N, %) | 9 (11.68%) | 17 (7.14%) | *p* = 0.20 |
|  | ARI (N, %) | 9 (11.68%) | 24 (10.08%) | *p* = 0.69 |
|  | QUE (N, %) | - | 24 (10.08%) | - |
|  | AMI (N, %) | - | 20 (8.40%) | - |
|  | ZIP (N, %) | - | 1 (0.42%) | - |
|  | HAL (N, %) | 0 | 22 (9.24%) | - |
|  | FLX (N, %) | 36 (46.75%) | - | - |
|  | ZUC (N, %) | 2 (2.59%) | - | - |
|  | LEV (N, %) | - | 3 (1.26%) | - |
|  | TIA (N, %) | - | 1 (0.42%) | - |
|  | CLZ (N, %) | - | 66 (27.73%) | - |

**Table S1.** Demographics and treatment characteristics of the study population.
