# Supplementary material for: Benzodiazepines and Mood Stabilizers in Schizophrenia Patients Treated with Oral versus Long-Acting Injectable Antipsychotics—An Observational Study
Source: Brain Sci. 2023 Jan 20;13(2):173. doi: 10.3390/brainsci13020173 (PMC9953951; doi:10.3390/brainsci13020173)
Supplement: Supplementary file 1 [file brainsci-13-00173-s001.zip › Table_S2_Mean antipsychotic dose and chlorpromazine equivalents.docx]

| Antipsychotic (type, formulation) | | | Number of cases | Mean dose (mg) | Oral dose equivalent (mg) | Chlorpro-  mazine equivalent (mg) | *p*-Value |
| --- | --- | --- | --- | --- | --- | --- | --- |
| **olanzapine** | LAI | | 5 | 480 (±  164.31) | 16 (±  5.47) | 320 | *p* = 0.66 |
|  | OAP | | 70 | 15 (±5) | 15 (±5) | 300 |  |
| **risperidone** | LAI | | 16 | 76.56 (±24.94) | 3.06 (±0.99) | 306 | *p* = 0.21 |
|  | OAP | | 31 | 3.58 (±1.50) | 3.58 (±1.50) | 358 |  |
| **aripiprazole** | LAI | | 9 | 400 | 20 | 266.66 | *p* = 0.22 |
|  | OAP | | 24 | 16.875 (±7.49) | 16.875 (±7.49) | 225 |  |
| **paliperidone** | LAI | | 9 | 180.55 (±  152.97) | 9.66 (±1.32) | 483 | *p* = 0.005 |
|  | OAP | | 15 | 7.4 (±1.91) | 7.4 (±1.91) | 370 |  |
| **quetiapine** | LAI | | - | - | - | - | NA |
|  | OAP | | 24 | 491.66 (±224.89) | - | 655 |  |
| **amisulpride** | LAI | | - | - | - | - | NA |
|  | OAP | | 20 | 500 (±247.08) | - | 290 |  |
| **ziprasidone** | LAI | | - | - | - | - | NA |
|  | OAP | | 1 | 120 | - | 200 |  |
| **haloperidol** | LAI | | - | - | - | - | NA |
|  | OAP | | 22 | 3.52 (±1.61) | - | 176 |  |
| **flupenthixol** | LAI | | 36 | 35 (±8.78) | - | 100 | NA |
|  | OAP | | - | - | - | - |  |
| **zuclopenthixol** | LAI | | 2 | 200 | - | 100 | NA |
|  | OAP | | - | - | - | - |  |
| **levomepromazine** | LAI | | - | - | - | - | NA |
|  | OAP | | 3 | 54.16 (±7.21) | - | 54.16 |  |
| **tiapridal** | LAI | | - | - | - | - | NA |
|  | OAP | | 1 | 100 | - | 100 |  |
| **clozapine** | LAI | | - | - | - | - | NA |
|  | OAP | Total | 66 | 272.72  (±126.51) | - | 272.72 |  |
|  |  | clozapine monotherapy | 53 | 297.64 (±128.56) |  | 297.64 |  |

**Table S2.** Mean antipsychotic dose and chlorpromazine equivalents.
