# Supplementary material for: Benzodiazepines and Mood Stabilizers in Schizophrenia Patients Treated with Oral versus Long-Acting Injectable Antipsychotics—An Observational Study
Source: Brain Sci. 2023 Jan 20;13(2):173. doi: 10.3390/brainsci13020173 (PMC9953951; doi:10.3390/brainsci13020173)
Supplement: Supplementary file 1 [file brainsci-13-00173-s001.zip › Table_S3_Table 3. Benzodiazepine types..docx]

|  | Diazepam | Alprazolam | Lorazepam | Clonazepam | Bromazepam | Nitrazepam | Cinolazepam |
| --- | --- | --- | --- | --- | --- | --- | --- |
| SGA-LAI (N, %) | 4 (10.25%) | 1 (2.56%) | 3 (7.69) | 2 (5.12%) | 1 (2.56%) | 1 (2.56%) | 1 (2.56%) |
| FGA-LAI (N, %) | 8 (21.05%) | 0 | 8 (21.05%) | 1 (2.63%) | 1 (2.63%) | 0 | 1 (2.63%) |
| OAP  (N, %) | 43 (18.06%) | 4 (1.68%) | 49 (20.58%) | 16 (6.72%) | 2 (0.84%) | 0 | 1 (0.42%) |

**Table S3.** Benzodiazepine types.
