# Supplementary material for: Benzodiazepines and Mood Stabilizers in Schizophrenia Patients Treated with Oral versus Long-Acting Injectable Antipsychotics—An Observational Study
Source: Brain Sci. 2023 Jan 20;13(2):173. doi: 10.3390/brainsci13020173 (PMC9953951; doi:10.3390/brainsci13020173)
Supplement: Supplementary file 1 [file brainsci-13-00173-s001.zip › Table_S4_Concomitant treatment by antipsychotic type..docx]

**Table S4.** Concomitant treatment by antipsychotic type.

| **Antipsychotic type** | **Formulation** | **MS (N, %)** | ***p*-Value** | **BZD (N, %)** | ***p-*Value** | **MSs and BZDs (N, %)** | ***p*-Value** |
| --- | --- | --- | --- | --- | --- | --- | --- |
| olanzapine | LAI (N = 5) | 1 (20%) | *p* = 0.77 | 0 | *p* = 0.10 | 0 | *p* = 0.42 |
|  | OAP (N = 70) | 18 (25.71%) |  | 25 (35.71%) |  | 8  (11.42%) |  |
| risperidone | LAI (N = 16) | 4 (25%) | *p* = 0.47 | 6 (37.50%) | *p* = 0.93 | 3  (18.75%) | *p* = 0.96 |
|  | OAP (N = 31) | 11 (35.48%) |  | 12 (38.70) |  | 6  (19.35%) |  |
| aripiprazole | LAI (N = 9) | 2 (22.22%) | *p* = 0.69 | 2 (22.22%) | *p* = 0.54 | 2  (22.22%) | *p* = 0.49 |
|  | OAP (N = 24) | 7 (29.16%) |  | 8 (33.33%) |  | 3  (12.50%) |  |
| paliperidone | LAI (N = 9) | 2 (22.22%) | *p* = 0.57 | 3 (33.33%) | *p* = 0.73 | 1  (11.11%) | *p* = 0.70 |
|  | OAP (N=15) | 5 (33.33%) |  | 4 (26.66%) |  | 1  (6.66%) |  |
