# Supplementary material for: Benzodiazepines and Mood Stabilizers in Schizophrenia Patients Treated with Oral versus Long-Acting Injectable Antipsychotics—An Observational Study
Source: Brain Sci. 2023 Jan 20;13(2):173. doi: 10.3390/brainsci13020173 (PMC9953951; doi:10.3390/brainsci13020173)
Supplement: Supplementary file 1 [file brainsci-13-00173-s001.zip › Table_S5_Monotherapy cases in the SGA-LAI group versus oral correspondents.docx]

**Table S5.** Monotherapy cases in the SGA-LAI group versus oral correspondents.

| Antipsychotic (type, formulation) | | Number of patients | Patients stabilized on monotherapy (N, %) | *p-*Value |
| --- | --- | --- | --- | --- |
| olanzapine | LAI | 5 | 4 (80%) | *p* = 0.18 |
|  | OAP | 70 | 32 (45.71%) |  |
| risperidone | LAI | 16 | 8 (50%) | *p* = 0.24 |
|  | OAP | 31 | 10 (32.25%) |  |
| aripiprazole | LAI | 9 | 5 (55.55%) | *p* = 0.25 |
|  | OAP | 24 | 8 (33.33%) |  |
| paliperidone | LAI | 9 | 4 (44.44%) | *p* = 0.83 |
|  | OAP | 15 | 6 (40%) |  |
