# Supplementary figures and images for: Benzodiazepines and Mood Stabilizers in Schizophrenia Patients Treated with Oral versus Long-Acting Injectable Antipsychotics—An Observational Study
Source: Brain Sci. 2023 Jan 20;13(2):173. doi: 10.3390/brainsci13020173 (PMC9953951; doi:10.3390/brainsci13020173)

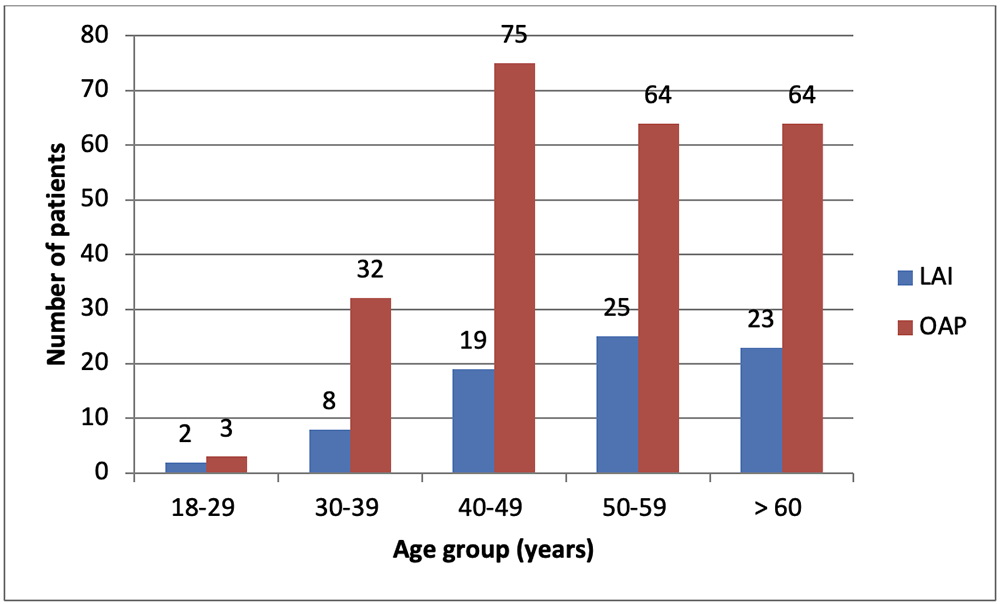

Supplement: Supplementary file 1 [file brainsci-13-00173-s001.zip › Figure_S1_Age_group_distribution_of_LAI_and_OAP.jpg]

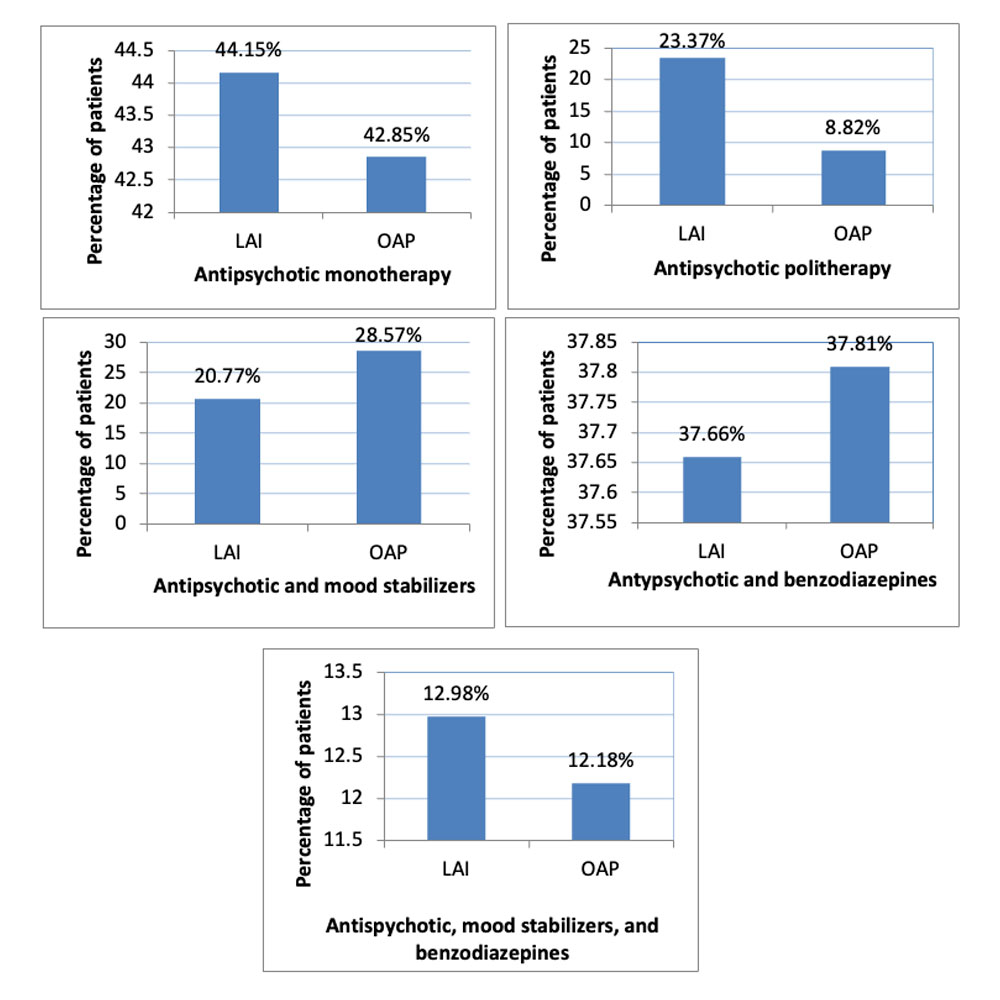

Supplement: Supplementary file 1 [file brainsci-13-00173-s001.zip › Figure_S2_Concomitant_treatment_LAI_versus_OAP.jpg]
